# Malaria chemoprophylaxis: cross-sectional study of use among air travellers departing from Accra, Ghana

**DOI:** 10.5281/zenodo.10756885

**Published:** 2017-02-28

**Authors:** Henry J.O. Lawson, Gerhard K. Ofori-Amankwah, Akye Essuman, Edwina B. Opare-Lokko, Charles Antwi-Boasiako, Andrew A. Adjei

**Affiliations:** 1 Korle-Bu Polyclinic, Korle Bu Teaching Hospital, Accra, Ghana; 2 Family Medicine Unit, Dept. of Community Health, School of Public Health, College of Health Sciences, University of Ghana, Accra, Ghana; 3 Dept. of Physiology, School of Biomedical and Allied Health Sciences, College of Health Sciences, University of Ghana, Accra, Ghana; 4 Office of Research, Innovations and Development, University of Ghana, Accra, Ghana

## Abstract

**Background:**

Malaria is the most common life-threatening infectious disease among travellers and chemoprophylaxis is recommended. The overall effectiveness, medication types and cost of malaria chemoprophylaxis in Accra are not well documented. This study investigated the use of chemoprophylaxis for malaria prevention in air travellers departing from Kotoka International Airport (KIA) in Accra, Ghana.

**Materials and methods:**

A cross-sectional study was conducted in the departure lounge of the KIA between February and May 2012. A total of 424 respondents voluntarily completed a semi-structured questionnaire, which included socio-demographic characteristics, duration of stay, nationality, country of permanent residence, chemoprophylaxis used, number of doses missed, cost and side effects experienced, and cost of treatment.

**Results:**

The mean age of respondents was 37 ± 0.84 years with a male:female ratio of 1.2:1.The mean duration of stay in Ghana was 47.9 days [SD 56.8] and 73.5% had made one trip to the country in the preceding year. Of the respondents, 50.7% were from Europe, 24.1% from North America and 17.5% from Africa. The most popular malaria prevention method used was prophylactics (37%) with atovaquone/proguanil used most frequently (34.9%), followed by mefloquine (11.6%) and doxycycline (7.8%). Compliance was high: 73.8% of respondents did not miss a single dose. The most commonly reported side effects were dreams, abdominal discomfort and headaches. Malaria incidence was 7.1% with 80% of them receiving treatment in a hospital or clinic; incurring a cost of up to $30 to treat a person.

**Conclusions:**

Most air travellers from Accr a take atovaquone/pr oguanil. Malaria incidence was low and most travellers were compliant with their chemoprophylaxis with very few side effects. The cost of chemoprophylaxis is low and is thus recommended for all travellers to Accra, Ghana.

## 1 Introduction

Malaria is a life-threatening disease caused by parasites transmitted to people through the bite of an infected female *A nopheles* mosquito [1]. About 3.3 billion people – ca. half the world's population - remain at risk of malaria and close to 200 million cases and half a million deaths are reported annually [2]. Malaria is preventable and curable and increased control efforts have dramatically reduced its burden in many places. Between 2000 and 2015, *Plasmo-dium falciparum* infection prevalence in endemic Africa halved and the incidence of clinical disease fell by 40%. It is estimated that interventions have averted 663 (542–753 range interval) million clinical cases since 2000. Insecticide-treated nets, the most widespread intervention, were by far the largest contributor (68%) to the cases averted [3]. Sub-Saharan Africa carried a disproportionally high share of the global malaria burden. In 2015, the region was home to 88% of malaria cases and 90% of all malaria deaths [1]. Malaria is the most common life-threatening infectious disease among travellers [4]. This makes malaria an important health risk for visitors to tropical areas where malaria is endemic. This fact is given further credence with the increasing number of travellers across all continents making malaria a very important disease in travellers to Accra, an endemic region for malaria.

The risk of malaria for travellers varies from region to region and depends on the intensity of transmission, the duration of stay in endemic areas, mode of travel, and efficacy of preventive measures [2]. Malaria is a major problem for Europeans when travelling to endemic regions. Between 10 and 15 million travellers visit malarious areas each year and 12-15 thousand cases are introduced in Europe afterwards, especially from African countries. For instance, in Romania, where malaria was eradicated in 1963, approximately 20 imported cases were detected annually during the period 1980-2007 [5]. Two-thirds of all *falciparum* malaria cases reported in the United Kingdom are acquired in West Africa [6,7]. The number of imported malaria cases is reported to have increased in China since 2008 [8]. The intensity of transmission depends on factors related to the parasite, the vector, the human host, and the environment. The long lifespan and strong anthropophilic biting habits of African vector species is an important reason why most of the world's malaria cases are in Africa [1]. Transmission is also known to vary in different communities within the same setting [9].

The increase in international travel, together with the large influx of immigrants from malaria-endemic countries, has had a significant impact on malaria cases in developed countries. Between 2001 and 2010, 45 countries in the European region reported a decline in imported malaria cases and deaths, possibly due to malaria control activities in endemic countries [10].

As international travel increases, there is a corresponding increase in the need to ensure that travellers are protected from diseases such as malaria. As part of preventive strategies to combat the disease, malaria chemoprophylaxis recommended to prevent *P. falciparum* infections in travellers to Ghana [1]. Malaria chemoprophylaxis is, however, problematic since there are issues with compliance due to fear of perceived and actual harmful side effects. Moreover, some patients lose confidence after previous experience of episodes of malaria despite adequate compliance with chemoprophylaxis. This has been reported for atovaquone/proguanil [11], mefloquine [12] and doxycycline [13]. Finally, there is scientific evidence to support the fact that immunity to malaria develops slowly in most naturally-exposed populations with protection against the parasite lagging behind protection against symptoms of the disease. Recent studies have, however, shown that even a single malaria episode induces robust cellular re-call responses to both parasite stages, persisting at almost undiminished levels at least 14 months post infection and involving both adaptive and innate compartments [14].

The use of chemoprophylaxis in air travellers to Accra is not well studied. There is scanty data on the number of air travellers to Accra that use malaria chemoprophylaxis as well as the level of compliance amongst these. The overall effectiveness, medication types and cost of malaria chemoprophylaxis are not known. The number of air travellers to Accra that develop malaria during their stay is also not well documented; moreover the economic impact of malaria in air travellers has not been studied in Ghana.

Wide gaps exist in knowledge regarding chemoprophylactic agents used by air travellers from Accra. The absence of such information denies travellers and health workers the opportunity to plan and manage patients with reduced or no immunity to malaria during their stay in endemic regions such as Ghana. This study documented these practices in air travellers, as well as provided the foundation for policy decisions on the prevention of malaria among air travellers from Accra.

## 2 Materials and methods

A cross-sectional study was conducted from February to May 2012, in the departure lounge of Kotoka International Airport (KIA), Accra. Most travellers from outside Africa enter Ghana through this airport. In 2006, the airport served 1.083.431 passengers and this figure increased to 1.8 million in 2012 [15].Thirty-one passenger and four cargo airlines use KIA regularly. This excludes personal and chartered flights. Twelve of the passenger airlines fly directly to non-malaria endemic destinations in Europe and North America. There are four boarding gates and one terminal at the airport. The 3rd and 4th boarding gates have a capacity for 170 travellers [16].

Access to the restricted departure lounge to administer questionnaires to travellers leaving Accra for countries where malaria is not endemic was granted by the Ghana Airports Company Ltd. All consenting travellers that had resided outside malaria-endemic regions for at least six consecutive months and had stayed in Ghana for at least two consecutive weeks were included in the study. Non-English and non-Ghanaian language speakers were excluded. Random sampling was used to select 10-12 respondents each day, at the final checkpoint before entering the departure lounge, until the sample size was completed. Passengers were usually seated in rows in the departure lounge. On sampling days, the principal investigator would direct three to four researchers to select alternate rows. Each row has four seats. On each row the researcher began with the first passenger to the right to seek eligibility for the study in terms of having stayed in the endemic region for at least two weeks. If this was affirmative, the consent was discussed. Consenting passengers were enrolled in the study and asked to complete the questionnaire on their own. For those with challenges in English language, guidance was given in local dialects. The team moved together to the five different departure lounges each night depending on the departure times of the various flights. Data collection was stopped at least 30 minutes before departure to allow the passengers time before boarding their aircrafts. Consent for participation was documented by signing an informed consent form. A copy of this form was given to each participant. Recruitment was done on all days of the week to ensure that the majority of the departing airlines were included. After completion of each workday, questionnaires were kept by the principal investigator and locked in a filing cabinet in the project office the following morning.

Ethical approval for this study was granted by the Ethical and Protocol Review Committee of the University of Ghana School of Medicine and Dentistry, Accra, Ghana. Data were analysed using SPSS, version 16.

## 3 Results and discussion

**Figure 1.**
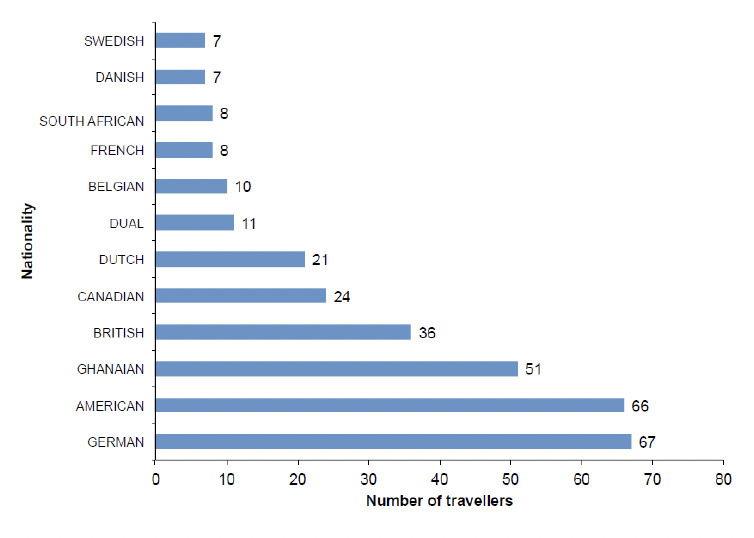
Top twelve nationalities (n=316) of respondents (n=424).

A total of 424 out of 507 respondents (non-response rate of 16.4%) completed the study; the mean age was 37 ± 0.84 years. The age range 18-30 years had the largest proportion of 35.1% followed by the 45-65 year group with 34.7% (Table 1). The male:female ratio was 1.2:1. Of the respondents, 58.7% had tertiary education and 49.6% were married. For occupation, 40.8% of the respondents indicated managerial or professional positions; 25.2% were students. The mean duration of stay was 47.9 days [SD 56.8] and approximately three-quarters of the respondents (73.5%) had made one trip to Ghana in the preceding year under while 13% (n=55) had made two trips. The average number of trips was 1.5 [SD 1.1] with one person that made twelve trips. 50.7% were Europeans, 24% were North Americans, 17.5% were Africans, and 19% were South Americans, Asians and Australians combined. The respondents had 51 nationalities with the top three being Germans, Americans and Ghanaians, in order of reducing numbers (Figure 1). For continent of permanent residence of respondents, 58.5% resided in Europe, 30.7% in North America, 6.1% in Africa, and 4.0% in South America, Asia and Australia/New Zealand combined (Table 1).

**Table 1.** Demographics and malaria prevention methods used by respondents (n=424).

Age (yrs)	N	%
18-30	149	35.1
31-44	86	20.3
45-65	147	34.7
>65	31	7.3
No Response	11	2.6
**Sex**		
Male	231	54.5
Female	193	45.5
**Continent of permanent residence**		
Europe	248	58.5
North America	130	30.7
Africa	26	6.1
Asia	8	1.9
Australia/New Zealand	5	1.2
South America	4	0.9
No response	3	0.7
**Marital status**		
Married (Opposite Sex)	203	47.9
Single	178	42.0
Divorced	19	4.5
No response	16	3.8
Separated	7	1.7
Married (same sex)	1	0.2
**Occupation**		
Professionals	130	30.7
Students	107	25.2
Managers	43	10.1
Service and sales workers	30	7.1
No response	29	6.8
Skilled agric, forestry and fishery workers	17	4.0
Craft and related trades workers	16	3.8
Elementary occupations	15	3.5
Retired/Pensioner	11	2.6
Technicians and associate professionals	11	2.6
Clerical support workers	9	2.1
Plant and machine operators, & assemblers	6	1.4
**Level of education**		
None	2	0.5
Basic	17	4.0
Secondary	88	20.8
Tertiary	249	58.7
Other	29	6.8
No response	39	9.2
**Cost of treating malaria (in US $)**		
1-5	125	65.4
6-10	47	24.6
11-15	15	7.9
16-20	3	1.6
>20	1	0.5
**Type of malaria prevention methods used**		
Other	6	1.4
Clothing	18	4.2
Bednet	20	4.7
Insecticides	26	6.2
Not Applicable	50	11.8
Prophylaxis	139	32.8
No Response	165	38.9

**Figure 2.**
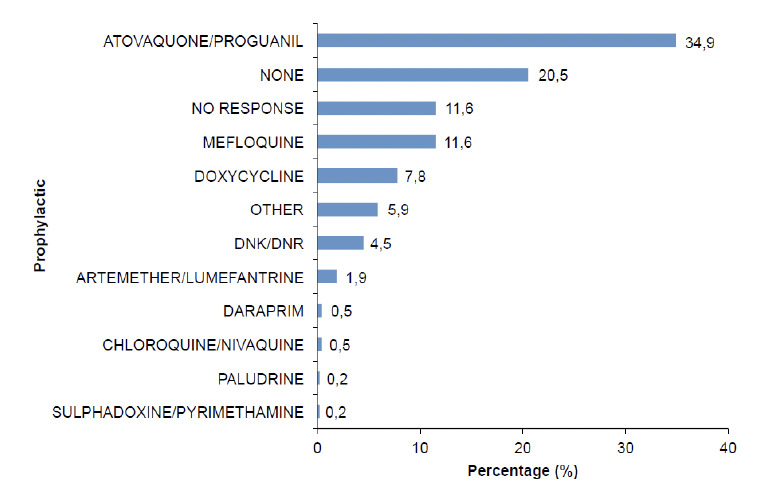
Malaria chemoprophylaxis taken during current trip (n=424).

Of the respondents, 49.1% reported using some form of malaria prevention. Methods used were prophylactic medication (32.8%), insecticides (6.2%), bednets (6.2%), clothing (4.2%) and others (1.4%) (Table 1). For prophylaxis, 34.9% used atovaquone/proguanil, 11.6% mefloquine, 7.8% doxycycline, 1.9% used chloroquine, Daraprim or sulphadoxine-pyrimethamine; and 5.9% used ‘other’ medication, which consisted of injections, vaccines, vitamin B and analgesics (e.g. naklofen duo) (Figure 2). The latter is a non-steroidal anti-inflammatory drug containing diclofenac sodium. It is noteworthy that 20.5% of the respondents admitted to the inappropriate use of artesunate/lumefantrine for prophylaxis even though it is only licensed for treatment.

**Figure 3.**
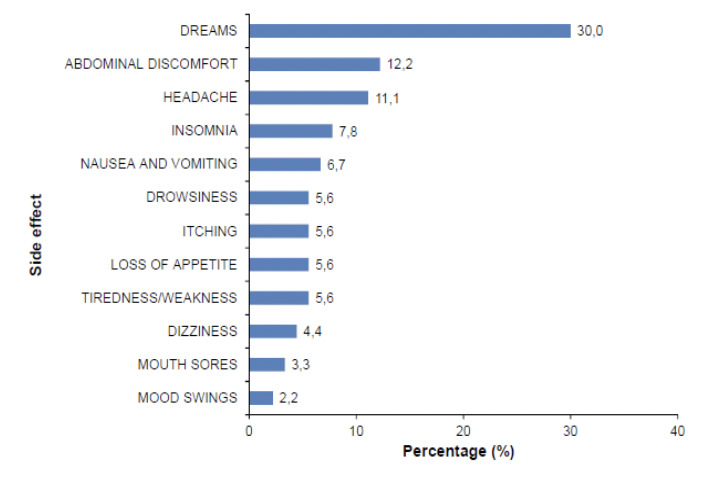
Side effects reported by travellers on malaria prophylaxis (n=90).

Out of 287 (67.6%) respondents who commented on side effects, one-third (31.4%) had experienced any. Dreams constituted 30% of side effects experienced which were described variably as being bad, funny, live, strange, vivid or weird. Other side effects recorded in order of decreasing frequency were abdominal discomfort, headache, insomnia, nausea and vomiting, drowsiness, itching, loss of appetite, tiredness/weakness, dizziness, mouth sores and mood swings (Figure 3).

Of respondents on prophylactic medication, 73.8% reported not missing any doses, 25.7% had missed 1-2 doses whilst the rest (3.5%) had missed up to 5 doses. For those that changed their medication, the most frequent reasons were ‘side effects’ (27.1%), ‘doctor’s advice’ (25.4%), ‘too many doses’ (18.6%) or ‘too many tablets’ (3.4%).

More than half (58%) of the subjects had never had malaria although 30 of them had had malaria on this trip, giving a malaria incidence rate of 7.1% in this cohort. On return to their countries of permanent residence, 22.5% of respondents had made arrangements to see a doctor. On cost of treating malaria, 65.4% spent less than $6; 32.5% spent $6-15, and 2.1% spent more than $15 (Table 1).

This study examined the chemoprophylaxis practices of travellers leaving Ghana by air. Air travellers from Accra who participated in the study were predominantly young and highly educated and as such much was expected from them in terms of basic knowledge on malaria prevention.

The majority of respondents originated from Europe. Studies conducted in Europe on malaria in travellers have consistently reported that travel to West Africa carries the highest risk of acquiring malaria, especially those returning from visits to friends and relatives (VFR) [10,17]. This adds credence to the fact that travellers need protection from acquiring malaria when visiting Ghana. The duration of stay was significantly correlated with the use of a malaria prevention method (p<0.001). This is supported by a study conducted by Schlagenhauf and Peterson [18] who reported that the risk of malaria for travellers is directly related to the duration of stay in an endemic area. Presumably, even though the duration of stay increases the duration of potential exposure to the disease, movement within the country and other factors also likely play a role [10].

Many travellers visiting African countries are VFRs. These VFRs are less likely to seek pre-travel health advice and have a lower tendency to use preventive measures compared to travellers with other travel purposes, such as tourism [14-16]. VFRs heading to West African countries have a high risk of contracting malaria [19]. People travelling for this purpose were at significantly higher risk of malaria than other travellers and were less likely to report the use of any chemoprophylaxis (odds ratio of reported chemoprophylaxis use 0.23, 95% confidence interval 0.21 to 0.25) [16]. This is in sharp contrast to the findings of the present study because we found that 37% of the travellers were on chemoprophylaxis and the majority of these (73.8%) had not missed a single dose. In a more recent study, Weiten *et al.* [19] reported that a higher percentage (53.9%) had started chemoprophylaxis, 60.4% had bought chemoprophylaxis and 67.5% had obtained pre-travel advice [19]. This may be due to increased awareness globally about the need to take prophylaxis to prevent malaria, availability of new drugs with fewer side effects, the educational status of this cohort and reduced malaria prevalence in endemic countries. It is important to recognize that VFRs are far from a homogenous group and additional research into the nature and extent of variation in knowledge, attitudes, and behaviours relating to prevention of malaria is required [20].

This study reports a malaria incidence of 7.1%. Behrens *et al.* [17] found a declining incidence of malaria with increasing travel to West Africa from the United Kingdom in 2008. They reported an annual decrease of 7.9%. This is higher than rates recorded for the Indian subcontinent (1.4% to 4.6%) [21]. This may be due to the fact that the *Plasmodium falciparum* is more virulent than the other *Plasmodium* species found in the Indian sub-continent. A cohort survey in Dutch travellers suggests an annual 1.8% increase in protection rates against malaria coinciding with an annual 2.5% decrease in intended risk-seeking behaviour [22].

The majority (73.8%) of those taking prophylactic medication did not miss any dose. This finding is similar to a study conducted on a cohort of French soldiers returning from Ivory Coast in 2007. They were randomized into two groups – one received a daily short message service (SMS) reminder message via mobile device to remind them to take their malaria chemoprophylaxis, and to assess the impact of the daily reminder SMS on chemoprophylaxis compliance. The control group did not receive any SMS alerts but were also expected to complete 28 days of daily 100 mg of doxycycline. Interestingly, compliance did not vary significantly between the two groups across the compliance indicators. This means that with the right motivation, travellers can be compliant with malaria prophylaxis medications [23].

This is in keeping with findings by McCarthy and Coyle [21] who interviewed 11 malaria experts. They reported that travellers are more likely to take prophylactic medication if there are no or mild adverse events and were least tolerant of mild sequelae from malaria and severe drug related events.

Studies have shown that atovaquone/proguanil is the chemoprophylactic agent that has the least adverse events (both mild and severe) compared to mefloquine and doxycycline. Doxycycline on the other hand is safer than mefloquine [24]. This study supported the leading role of atovaquone/proguanil but did not support a preference for doxycycline over mefloquine. The decision whether to use malaria prevention was significantly related to continent of birth (p=0.005); continent of nationality (p=0.034); continent of permanent residence (p=0.001); and marital status (p=0.02). It was, however, not significantly associated with occupation (p=0.455) nor the level of education (p=0.577). When choosing a malaria prevention method, the following were found to be significantly associated – continent of birth (p<0.001); continent of nationality (p<0.001); continent of permanent residence (p=0.012); marital status (p<0.001); occupation (p=0.003) and level of education (p<0.001). Additionally, it was significantly associated with the time when respondents last had malaria (p=0.009).

The majority (68.6%; n=287) of respondents that commented on side effects had not experienced any. The commonest side effect was dreams (30%), which compares favourably with other studies reporting dominating neuropsychiatric side effects [25]. Photosensitization, which is an uncommon but sometimes severe and prolonged side effect of using doxycycline, was not recorded in the present survey.

The majority (76.7%) of patients that contracted malaria underwent laboratory testing once and 50% conducted a repeat laboratory test. Twenty-four (80%) respondents had been treated in a hospital or clinic. The remaining six were either treated in pharmacies (n=3) or practiced self-medication (n=3). Ten of them had been admitted but only two had received intravenous infusions or intramuscular injections. The majority (n=16) had completed data on the cost of care with 68.8% (n=11) of them spending up to $30 on treatment.

More than half of the respondents (62.2%) spent $1-5 per day on chemoprophylactic medication and up to 85.6% spent less than $15 per day. There was a high non-response rate (55%) for this question because most respondents collected the medication with various forms of health insurance and thus did not know the actual costs involved. For travellers living in Europe and other developed countries, these costs are not prohibitive and thus considered a worthy investment to protect against malaria. Migrants in many European countries often live in areas of high socioeconomic deprivation and money spent on travel may take priority over the expense of chemoprophylaxis [10]. Some migrants may be unwilling to engage with the formal health care services. Ten per cent of respondents from the Netherlands did not have health insurance, and were assumed to reside there illegally [20].

Malaria episodes were significantly related to the continent of birth (p=0.010); number of missed doses of malaria prophylaxis medication (p=0.005) but not with continent of nationality (p=0.909) or continent of permanent residence (p=0.939). It was also not significantly associated with marital status (p=0.958); occupation (p=0.717); level of education (p=0.950) and which malaria prevention method used (p=0.226). Malaria attacks were also not significantly associated with the number of trips to Accra (p=0.999).

The age of the respondents was significantly associated with number of doses missed (p<0.001) and whether medication has been changed (p<0.001).

## 4 Conclusion

Chemoprophylaxis for malaria is practiced by the majority of air travellers from Accra. Atovaquone/proguanil is the most popular choice with minimal side effects recorded. There was a low incidence of malaria episodes in this cohort. Costs for both medications for prevention and treatment were minimal.
